# Emerging Materials for Additive Manufacturing

**DOI:** 10.3390/ma16010127

**Published:** 2022-12-23

**Authors:** Swee Leong Sing, Wai Yee Yeong

**Affiliations:** 1 Department of Mechanical Engineering, National University of Singapore, 9 Engineering Drive 1, Singapore 117575, Singapore; 2 Singapore Centre for 3D Printing, School of Mechanical & Aerospace Engineering, Nanyang Technological University, 50 Nanyang Ave, Singapore 639798, Singapore

Additive manufacturing (AM) has grown and evolved rapidly in recent years. There are many exciting research and translational works in many areas of application, such as biomedical [1,2], aerospace [3,4] and electronics [5,6,7]. These advancements are typically coupled with materials development, which has resulted in more functionalities added to 3D printed parts, such as multi-material fabrications [8,9,10] and integration with machine learning or digital twins [11,12,13]. Such enhancements in functionalities have enabled the evolution of AM from a rapid prototyping tool to an actual manufacturing solution.

In this Special Issue, state-of-the-art research and review articles on emerging material systems for AM are collected, with a focus on the process–structure–properties relationships. In total, two reviews and thirteen original research articles are included. In their review article, Minasyan and Hussainova discussed the recent developments of ceramic particulate-reinforced aluminium alloys produced by laser powder bed fusion [14], while Hou et al. elaborated the use of monitoring systems for powder bed fusion processes with a focus on metals in their comprehensive review [15]. For original research, Gatões et al. studied the fabrication of different stainless steels using selective laser melting, a type of laser powder bed fusion technique [16]. In their study, Mally et al. benchmarked the mechanical properties of ferritic steels produced by selective laser melting with relevant forged parts [17]. Using selective laser melting as well, Koh et al. studied the fabrication of silica-reinforced steel matrix nanocomposites [18]. Lim et al. studied the bone conduction capacity of highly porous titanium scaffolds with different designs produced by selective laser melting [19]. Chen et al. studied the effect of laser scanning speed on the microstructure and mechanical properties of K418 nickel-based alloy produced by laser powder bed fusion [20]. Böhm et al. evaluated the feasibility of using a mixture of two aluminium alloys to eliminate solidification cracks formed during laser powder bed fusion [21]. Chen et al. studied the fabrication of bimetallic structures using TiNi-based shape memory alloy by laser-directed energy deposition [22]. Also using laser-directed energy deposition, Menon et al. attempted to quantify the process using multi-fidelity surrogate-based process mapping [23]. Hein et al. studied the effect of heat treatment on metastable β titanium alloy produced by laser powder bed fusion [24]. Romani et al. studied the metallization of recycled glass fibre-reinforced polymers that are processed by UV-assisted 3D printing [25]. Hailu et al. studied the effect of structure design on the performance of functionally graded materials produced by the MultiJet Fusion technique [26]. Marczyk et al. analysed the use of concrete–geopolymer hybrids reinforced with aramid roving for 3D concrete printing [27]. Yao et al. evaluated the feasibility of colour 3D printing by studying the pigment penetration in powder-based additive manufacturing [28].
